# Development of Rapid Detection Methods for *Fusarium oysporum* f. sp. *melonis* in Melon Seeds

**DOI:** 10.3390/ijms25105371

**Published:** 2024-05-14

**Authors:** Tsai-De Chang, Ya-Zhen Xu, Yu-Fen Wang, Xing-Ru Wang, Shang-Han Tsai, Zhong-Bin Wu, Yuh Tzean, Ying-Hong Lin

**Affiliations:** 1 Department of Plant Medicine, National Pingtung University of Science and Technology, Pingtung 91201, Taiwan; b10127038@gmail.com (T.-D.C.); koji860206@gmail.com (Y.-Z.X.); b10527027wang@gmail.com (Y.-F.W.); ella40332@gmail.com (X.-R.W.); 2 Bachelor Program in Scientific Agriculture, National Pingtung University of Science and Technology, Pingtung 91201, Taiwan; tsaihank@mail.npust.edu.tw; 3 Department of Horticulture and Landscape Architecture, National Taitung Jr. College, Taitung 95045, Taiwan; zhong.binwu@ntc.edu.tw

**Keywords:** rapid DNA extraction method, polymerase chain reaction (PCR), Probe-qPCR, disease management

## Abstract

Melon (*Cucumis melo* L.) is a global commercial crop that is sensitive to seed-borne wilt infections caused by *Fusarium oxysporum* f. sp. *melonis* (Fom). To address the challenge of detecting Fom contamination, we designed a probe-based real-time PCR method, TDCP2, in combination with rapid or column-based DNA extraction protocols to develop reliable molecular detection methods. Utilizing TDCP2, the detection rate reached 100% for both artificially Fom-inoculated (0.25–25%) and pod-inoculated melon seeds in conjunction with DNA samples from either the rapid or column-based extraction protocol. We performed analyses of precision, recall, and F1 scores, achieving a maximum F1 score of 1 with TDCP2, which highlights the robustness of the method. Additionally, intraday and interday assays were performed, which revealed the high reproducibility and stability of column-based DNA extraction protocols combined with TDCP2. These metrics confirm the reliability of our developed protocols, setting a foundation for future enhancements in seed pathology diagnostics and potentially broadening their applicability across various Fom infection levels. In the future, we hope that these methods will reduce food loss by improving the control and management of melon diseases.

## 1. Introduction

Melon (*Cucumis melo* L.) is a popular fruit worldwide, recognized for its high carotene and sugar content [1]. According to the data provided by the Food and Agriculture Organization (FAO) of the United Nations in 2022, global melon production reached 28,558,068.57 tons, spanning a harvested area of 1,062,501 hectares [2]. In Taiwan, despite the smaller production scale, 27,364 tons of melon were produced across 2134 hectares in 2022 [3]. The melon production in Taiwan for 2021 was TWD 2,116,634, ranking it as the fourth-largest fruit vegetable crop in Taiwan [3].

In the cultivation of melon crops, the fungus *Fusarium oxysporum* f. sp. *melonis* (Fom), known to cause severe vascular wilt, is one of the most significant pathogens affecting melon worldwide [4]. Fom is classified into four physiological races based on the specific resistance gene (S) they overcome: race 0, race 1, race 2, and race 1.2. Furthermore, race 1.2 is subdivided into race 1.2y (yellowing) and race 1.2w (wilting) [4,5]. The disease manifests in several ways, with seedlings showing symptoms such as hypocotyl rot and damping-off, while adult plants may exhibit one-sided wilt. As the disease progresses, leaves may turn completely yellow, wilt, and eventually die [6]. Other notable symptoms include the appearance of brown necrotic streaks and gummy exudates on the basal stems of the melon plant [7]. The impact of Fom on melons can be profound, with potential losses reaching up to 100%, marking it one of the most devastating diseases for this crop [4].

The spread of Fom may occur through seeds contaminated with the pathogen, dispersing to various areas [8,9]. Therefore, an ideal seed assay that is sensitive, specific, rapid, robust, inexpensive, and straightforward for the detection of Fom is pertinent [10]. If a seed assay lacks fidelity, it can produce incorrect results and delayed decision-making, potentially leading to disease spread and economic losses. Moreover, an overly complex assay may require significant resources and expertise, potentially causing implementation challenges and errors, especially in resource-limited settings. Therefore, these characteristics are essential for effective and efficient disease control in agricultural practices [11,12,13,14].

Molecular methods offer advantages over classical detection methods because they provide sensitive, reliable, and rapid quantification [15]. Efficient seed health testing methods can assist in preventing the long-distance spread of this pathogen via inoculated seeds [16]. Molecular methods, including polymerase chain reaction (PCR)-based techniques, have been frequently used to identify and detect plant pathogens, including *Fusarium* species [17]. Techniques such as conventional PCR (cPCR) [4,18], loop-mediated isothermal amplification (LAMP) [19], SYBR-qPCR [18,20], and Probe-qPCR [8,18], have been developed for the rapid and specific detection of Fom. Recently, unique primers designed to distinguish different pathogens have shown substantial benefits [21]. Sequences from highly conserved genes, including translation elongation factor 1α (TEF-1α), calmodulin, and beta-tubulin serve as effective genetic markers for differentiating fungal species, including *Fusarium* spp. [4].

Molecular techniques, including the conversion of Random Amplified Polymorphic DNA (RAPD) into Sequence Characterized Amplified Regions (SCARs), enhance the reproducibility and reliability of pathogen detection. This method has been instrumental in identifying different races of *F. oxysporum*. In a previous study, Luongo et al. [4] developed a specific Fom race 2 primer set based on a unique marker identified using RAPD. Similarly, López-Mondéjar et al. [8] designed primers and probes based on Fom-SCAR fragments, while Haegi et al. [20] and Edel et al. [22] designed primers for TEF-1α and ribosomal DNA genes, improving Fom detection in various plant tissues. However, the development of methods for reliably detecting Fom in naturally infected seeds lags behind. Current techniques like SYBR-qPCR and Probe-qPCR have shown potential in detecting naturally infected seeds of other *Fusarium* species [23,24], but similar advances for Fom are scarce. Chang et al. [18] made some progress by detecting up to 50% of artificially inoculated Fom seeds, yet no validated method exists for naturally infected seeds, highlighting a critical research gap. Additionally, the reproducibility of these detection methods is critical, often assessed through intraday and interday assays. Studies by Gao et al. [24] and Lin et al. [21] have emphasized the necessity of consistent and reliable assays to ensure valid results across different time points. Despite these efforts, a standardized method to assess the reproducibility of Fom detection in naturally-infected seeds is still needed.

An ideal DNA extraction method should efficiently yield high-quality DNA, devoid of PCR-inhibitory substances, while also being cost-effective, rapid, and scalable [25]. Traditional organic reagent extraction methods are effective but time-consuming, involving the use of hazardous solvents such as chloroform and phenol. These pose risks to both the user and the environment [26,27]. In recent times, column purification has become the primary method for DNA extraction. [21,28]. This technique employs spin columns filled with solid-phase nucleic acid binding materials, facilitating the easy binding, washing, and elution of nucleic acids during the purification process [28]. While this approach can efficiently yield high-quality DNA for further DNA amplification, it is designed for one-time use and can be more costly than traditional organic reagent extraction methods.

To overcome these limitations, this study introduces two molecular detection methods using Probe-qPCR, combined with both rapid and column-based DNA extraction protocols. The primary objective of this study is to develop and optimize rapid and accurate molecular detection methods for Fom in melon seeds. This includes establishing a rapid DNA extraction method that significantly reduces the time required to obtain DNA from melon seeds. Additionally, we aim to enhance the precision of molecular assays to minimize false negatives and false positives in identifying Fom infections in melon seeds. A further objective is to validate the effectiveness of these methods through laboratory tests on both artificially and pod-inoculated seeds. These efforts are directed towards facilitating quicker diagnostic responses that could potentially reduce the spread and economic impact of the disease in melon cultivation.

## 2. Results

### 2.1. The Specificity Test of the Primers

RAPD markers successfully identified the Fom race 2 molecular marker. Based on this design, the Fom race 2 primer set Fa15f/Fa15R was chosen. Furthermore, using this primer set as a foundation, we designed the primer set TDCP2F/TDCP2R. We first tested the specificity of the previously described primer set Fa15F/Fa15R [4] and our designed primer set TDCP2F/TDCP2R to Fom using PCR followed by gel electrophoresis. In addition to Fom, the fungal pathogens tested include *F. oxysporum* f. sp. *tracheiphilum* (YHL-F003), *F. oxysporum* f. sp. *gladioli* (YHL-F019), *F. oxysporum* f. sp. *lactucae* (Fola, YHL-F031 and ATCC76616), *F. oxysporum* f. sp. *lilii* (YHL-F035), *F. oxysporum* f. sp. *lycopersici* (YHL-F042), *F. oxysporum* f. sp. *niveum* (ATCC62940), *F. oxysporum* f. sp. *cubense* (Foc, YJL-F040, ATCC38741, ATCC76243, and ATCC96285), *F. oxysporum* f. sp. *anoectochili* (YHL-F002), *F. oxysporum* (YJL-F056 and TDC-F012), *F. solani* (YJL-F055), *F. acuminatum* (YHL-F018), *F. verticilliodes* (YHL-F056), *Colletotrichum orbiculare* (Co, TDC-F013 and TDC-F014), and *Alternaria* sp. (TDC-F016). In the analyzed gel electrophoresis, only samples containing Fom displayed visible bands at 301 bp with primer set Fa15F/Fa15R and 114 bp with TDCP2F/TDCP2R. These findings demonstrate the specificity of reference primer Fa15F/Fa15R [4] and our designed primers, TDCP2F/TDCP2R, to Fom (Table 1).

### 2.2. The Sensitivity Test of the Primers

**Figure 1:**
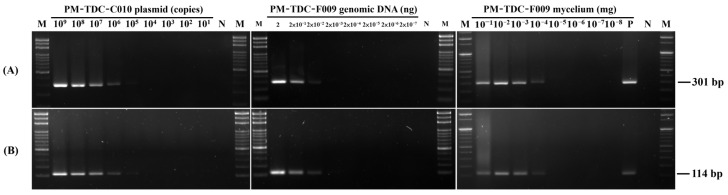
Detection sensitivity of conventional PCR assays using the primer sets Fa15F/Fa15R (**A**) and TDCP2F/TDCP2R (**B**). In these assays, serial dilutions of three types of samples, including standard DNA, genomic DNA, and mycelium were used as templates. DNA bands at positions corresponding to 301 bp (for Fa15F/Fa15R) and 114 bp (for TDCP2F/TDCP2R) are visible. For reference, a negative control (N), and a positive control (P), in which sterile double-distilled water (ddH_2_O) and 2 ng of Fom genomic DNA were used as the templates, respectively. A Gen-100 DNA ladder (M, GMbiolab Co. Ltd., Taichung, Taiwan) served as a reference for determining the size of the amplified DNA fragments.

**Figure 2:**
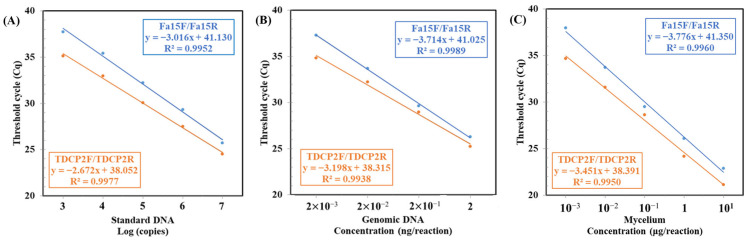
Detection sensitivity of Probe-qPCR assays with the primer sets Fa15F/Fa15R and TDCP2F/TDCP2R. Serial dilutions of three types of samples, including (**A**) standard DNA, (**B**) genomic DNA, and (**C**) mycelium were individually used as templates. Standard curves were generated by plotting the quantity of each of the three samples against their corresponding threshold cycle (Ct) values.

Upon establishing the specificity of both primer sets, we proceeded to evaluate their sensitivity. We utilized conventional PCR and Probe-qPCR to analyze various templates from Fom, including standard DNA (cloned fragment DNA), genomic DNA (gDNA), and mycelia. The sensitivity results for both primer sets (Fa15F/Fa15R and TDCP2F/TDCP2R) are displayed in Figure 1 and Figure 2. In conventional PCR, the sensitivity levels for detecting standard DNA, gDNA, and mycelia were 10^6^ copies (Figure 1A), 2 × 10^−2^ ng (Figure 1B), and 1 µg (Figure 1), respectively. Meanwhile, in Probe-qPCR, the sensitivity levels for detecting standard DNA, gDNA, and mycelia were 10^3^ copies (Figure 2A), 2 × 10^−3^ ng (Figure 2B), and 1 ng (Figure 2C), respectively. The TDCP2 Probe-qPCR yielded lower Ct values (Figure 2). These results suggest that both primer sets demonstrate similar levels of sensitivity.

These results indicate that the two primer sets (Fa15F/Fa15R and TDCP2F/TDCP2R) show comparable sensitivity in detecting Fom, both in conventional PCR and Probe-qPCR analyses. When detecting standard DNA, gDNA, and mycelia, the TDCP2 Probe-qPCR resulted in lower Ct values, suggesting its potential for greater efficacy in detecting lower quantities of templates. These data support the utility of both primer sets as effective tools for the detection of Fom (Figure 2).

### 2.3. Comparing the Reproducibility of the Molecular Detection Systems

To evaluate the reproducibility of molecular detection systems, the DNA samples were subjected to both conventional PCR and Probe-qPCR analyses, and the coefficients of variation (CV) were subsequently calculated (as detailed in Table 2). We analyzed four different samples at the same time points (intraday) and four replicates of the same samples in different days (interday), with the whole reproducibility assay conducted in experimental sets. Each intraday and interday sample was sampled three times for molecular detection. The assessment of reproducibility showed that the smaller the value, the smaller the coefficients of variation. This represents the better reproducibility of molecular detection systems [30]. As shown in Table 2, the detection reproducibility of conventional PCR for diagnosing Fom in the artificially inoculated seeds consistently remained lower than that of probe-qPCR regardless of the DNA extraction methods employed. In comparison, the TDCP2F/TDCP2R primer set demonstrated superior reproducibility in detecting Fom in artificially inoculated seeds at a 5% level, compared to the Fa15F/Fa15R primer set.

### 2.4. Comparing the Molecular Detection Results from the Different Extraction Systems

To compare the effectiveness of different molecular detection systems in seeds inoculated with varying levels of pathogens, DNA was extracted from 100 mg subsamples of 400 seeds artificially inoculated with Fom at levels ranging from 0% to 25%. DNA was extracted from these seeds using the rapid DNA extraction technique or a commercial spin column-based DNA extraction procedure and followed by either conventional PCR or Probe-qPCR (as detailed in Table 3 and Table 4). When employing column-based DNA extraction and analyzing with Probe-qPCR, the detection rates for 0.25% artificially Fom-inoculated seeds using the primers Fa15F/Fa15R and TDCP2F/TDCP2R were 77.78% and 100%, respectively (Table 3). In contrast, the detection rates using conventional PCR with the same primer pairs were only 33.3% for either primer sets. For samples extracted using the rapid DNA extraction method and analyzed with Probe-qPCR, the detection rates for 0.25% artificially Fom-inoculated seeds using the primers Fa15F/Fa15R and TDCP2F/TDCP2R were 0% and 100%, respectively (Table 4). The detection rates using conventional PCR with Fa15F/Fa15R and TDCP2F/TDCP2R primer sets were 33.3% and 22.2%, respectively (Table 4).

In the evaluation of artificially Fom-inoculated seeds at levels ranging from 0% to 25%, key metrics including precision (True positives/(True positives + False positives)), recall (True positives/(True positives + False negatives)), accuracy ((True positives + True negatives)/(True positives + False positives + False negatives + True negatives)), and F1 scores (the harmonic mean of precision and recall) were utilized to compare the performance of eight molecular detection protocols (Table 5). As indicated in Table 5, TDCP2 Probe-qPCR in combination with either of the DNA extraction methods, demonstrated superior results when detecting infection in seeds inoculated with various levels (0.25 to 25%) of Fom. The precision, recall, and accuracy of these two protocols were all equal to 1, surpassing the performance of the other four protocols (Table 5). This finding suggests that the application of TDCP2 Probe-qPCR, coupled with either of the two DNA extraction methods utilized in this study, allowed the most accurate detection of Fom in melon seeds among the evaluated protocols (Table 5). Moreover, TDCP2 Probe-qPCR in conjunction with either the column DNA extraction method or the rapid DNA extraction method showed the highest F1 scores (Table 5).

### 2.5. Molecular Detection Results of Pod-Inoculated Seeds

To evaluate the efficacy of either the previously published conventional PCR method (Fa15 cPCR) [4] or the newly designed high-stability molecular detection protocols of TDCP2 Probe-qPCR in the detection of pod-inoculated seeds, a peduncle inoculation procedure was conducted in a greenhouse to simulate natural infection. Utilizing column-based DNA extraction in conjunction with Probe-qPCR, the detection rates for the pod-inoculated seeds incubated for 0 to 21 days were consistently 100% (Table 6). In stark contrast, the detection rates with Fa15 cPCR were 0% for either of the DNA extraction methods. The detection rates for pod-inoculated seeds by employing rapid DNA extraction and Probe-qPCR varied depending on the day after incubation. The detection rate varied from 67% to 100% on different days after incubation (Table 6). These findings underscore that the detection efficacy of TDCP2 Probe-qPCR was markedly higher than that of Fa15 cPCR for pod-inoculated seeds. Notably, when TDCP2 Probe-qPCR was paired with column-based DNA extraction for detecting the pod-inoculated seeds at 0 days post-incubation, the detection rate reached 100% (Table 6). This outcome suggests that the seeds could be immediately tested without needing to amplify the pathogen levels through cultivation. While the detection rate using rapid DNA extraction was only 67%, conventional pathogen isolation methods yielded no detection at all, further emphasizing the superior sensitivity of the TDCP2 Probe-qPCR method.

## 3. Discussion

In the context of molecular diagnostics, the Random Amplified Polymorphic DNA (RAPD) technique, while initially useful, has faced challenges such as low reproducibility and high DNA quantity requirements. To overcome these limitations, specific target sequences from RAPD are often converted into Sequence Characterized Amplified Regions (SCARs), which significantly improve reliability and reproducibility, as noted in the existing literature [4]. These SCAR markers have been instrumental in identifying various formae speciales and races of *F. oxysporum*. For instance, Luongo et al. [4] utilized RAPD to pinpoint a molecular marker specific to Fom race 2, subsequently designing a specific primer set based on this marker. Similarly, López-Mondéjar et al. [8] crafted primers and probes derived from Fom-SCAR fragments to facilitate the detection of Fom-infected melon seedlings in greenhouse conditions. Further refining the approach, Haegi et al. [20] and Edel et al. [22] leveraged the nucleic acid sequences of TEF-1α genes and ribosomal DNA genes, respectively, to design primer sets that enhance the detection capabilities for Fom. These developments highlight a progressive shift towards more precise and dependable molecular diagnostics in plant pathology, underscoring the potential for such technologies to evolve and cater to specific pathogen detection needs in agricultural settings.

In this study, the specific primer pair TDCP2F/TDCP2R was designed to develop a rapid detection method for Fom-inoculated seeds. Probe-qPCR systems have been shown to be more sensitive than conventional PCR [31]. Within the Probe-qPCR system, the primer pair TDCP2F/TDCP2R, along with the probe TDCpr1, was designed to analyze standard DNA, genomic DNA, and Fom mycelium. When compared to the primer pairs Fa15F/Fa15R, the result showed that the combination of TDCP2F/TDCP2R with the probe TDCpr1 yielded a positive detection with a lower Ct value (Table 2). Previous reports also indicate that a short-amplified fragment primer combined with a probe results in a high reaction efficiency [32]. The slope of the qPCR standard curve represents this reaction efficiency, and some scholars have proposed a conversion method where the reaction efficiency equals 10^−1/slope^. Consequently, if the slope increases, the reaction efficiency will also increase, and the two values are proportional [33]. Furthermore, when using qPCR technology to detect Fom samples in this study, the overall results demonstrated that Probe-qPCR was more sensitive than conventional PCR, corroborating similar findings by Rodríguez et al. [31,34].

The intraday and interday analysis of melon seeds with a 5% artificial infection level primarily serves to evaluate the reproducibility of different primers and molecular detection methods when testing samples at various time points. It has previously been suggested that a coefficient of variation rate less than 25% is considered acceptable for qPCR [35]. In this study, the intraday and interday variation rates for Probe-qPCR with the newly designed primer pair TDCP2F/TDCP2R were found to be 0.78% and 1.5%, respectively (Table 2). These rates were lower than those of Fa15F/Fa15R, which exhibited intraday and interday variation rates of 3.78% and 8.35% (Table 2), respectively. This result indicates that the primer pair TDCP2F/TDCP2R provides the highest detection reproducibility among 5% Fom-inoculated seeds (Table 2).

Plants contain substances like polysaccharides, phenols, and proteins, all of which are common PCR inhibitors [36]. Melon seeds, composed of polysaccharides [37], proteins, and fatty acids [38], have high concentrations of polysaccharides and proteins that can affect the quality of extracted DNA [36,39]. In this study, the rapid DNA extraction method utilized is a crude extraction method that lacks a DNA purification step. As a result, these substances of melon seeds may inhibit qPCR, leading to suboptimal detection rates (Table 3 and Table 4).

The distribution of infested seeds within a seed lot may not always be uniform, and certain seeds might contain inoculum levels beneath the detection threshold. This can introduce variability in Fom infestation detection when assays are conducted via plating. Such variability in detecting infested seeds has been observed in other pathosystems as well [23]. In our study, although detection rates varied from 67 to 83% between 0 and 7 days post incubation (dpi), at longer incubation times (14 dpi), even using the rapid nucleic acid extraction method, Probe-qPCR successfully achieved a 100% detection rate (Table 6).

In this study, after performing detection on the artificially inoculated seeds, we calculated and compared the precision, recall, accuracy, and F1 score values (Table 5). In the future, we plan to conduct random sampling of imported and exported melon seeds. These seeds will be incubated in growth chambers to determine whether they carry Fom. Subsequently, the precision, recall, accuracy, and F1 score values will be calculated and compared, allowing for a comprehensive analysis of the detection method’s efficacy.

Our study conclusively demonstrates a consistent preference for Probe-qPCR over conventional PCR in terms of reproducibility and effectiveness. This is because when using the TaqMan probe fluorescence system for amplification in qPCR, primer pairs generating smaller amplified fragments tend to have a higher amplification efficiency than those generating longer fragments [40]. In our study, the amplified fragments of TDCP2F/TDCP2R are smaller than those of Fa15F/Fa15R, making them more resistant to the interference of PCR inhibitors. We also critically evaluated two different DNA extraction methods— rapid DNA extraction and commercial spin column-based extraction— and found that the latter shows better reproducibility and effectiveness, especially when paired with the TDCP2 Probe-qPCR method. The enhanced sensitivity of Probe-qPCR was particularly evident, asserting its advantage in both artificial (Table 3 and Table 4) and natural (Table 6) Fom contamination scenarios. The comprehensive assessment of reproducibility indicated that consistent results could be achieved with TDCP2 Probe-qPCR, lending credibility to its application in a real-world scenario. Our assessment was conducted across various contamination levels (from 0.25% to 25%). The TDCP2 Probe-qPCR, combined with both DNA extraction methods, showed superior performance, validating its adaptability across different infection intensities (Table 4).

As detailed in Table 6, the natural contamination experiment replicated a real-world scenario, emphasizing the applicability of the findings. The TDCP2 Probe-qPCR’s detection efficacy of 100% for pod-inoculated seeds further underscores its superiority. The marked difference between the detection rates of TDCP2 Probe-qPCR and Fa15 cPCR (0%) highlights the necessity for careful method selection in real-world applications. Moreover, our findings showed that TDCP2 Probe-qPCR allows for immediate seed testing without necessitating pathogen amplification, enhancing its practical allure. These discoveries hold the potential to influence quality control practices in agriculture, potentially setting a new benchmark for detecting Fom in melon seeds. Understanding why some methods (e.g., Fa15 cPCR) failed and validating these findings across diverse conditions could be valuable future research directions.

## 4. Materials and Methods

### 4.1. Pathogen Isolates and Growth Condition

The Fom isolates utilized in this study were confirmed through a pathogenicity test on their original hosts and are listed in Table 1. Additionally, the genomic DNA (gDNA) from a variety of other fungal pathogens was employed for comparison. In these experiments, Fom isolates no. PM-TDC-F006~F011, PM-YJL-F053~F054 and others, including pathogenetic strains no. PM-TDC-F012~F014, PM-TDC-F016, PM-YHL-F002~F003, PM-YHL-F018~F019, PM-YHL-F031, PM-YHL-F035, PM-YHL-F042, PM-YHL-F056, PM-YJL-F040, PM-YJL-F055, and PM-YJL-F056 were collected in Taiwan from 2001 to 2024. *Fusarium oxysporum* f. sp. *lactucae* strain no. ATCC76616, *F. oxysporum* f. sp. *niveum* strain no. ATCC62940, *F. oxysporum* f. sp. *cubense* strain no. ATCC38741, ATCC76243, and ATCC96285 were purchased from the American Type Culture Collection (Manassas, VA, USA). For growth conditions, a single spore culture of each tested Fom and *Fusarium* sp. isolate was cultivated on a Nash−PCNB plate. The plate contained 1.5% peptone, 2% agar, 0.1% KH_2_PO_4_, 0.05% MgSO_4_·7H_2_O, 0.1% pentachloronitrobenzene, 0.03% streptomycin, and 0.1% neomycin [41]. In parallel, a single spore culture of other fungal pathogens was nurtured on a potato dextrose agar (PDA) plate, composed of 200 g/L of potato extracts, 1% glucose, and 2% agar. Following a 7-day incubation period, the mycelia were harvested from the plates for further DNA isolation. The controlled growth conditions and uniform collection method ensured consistency across the various strains, establishing a solid foundation for subsequent comparative analysis.

### 4.2. Primer and TaqMan Probe Design

The specific primer set Fa15F/Fa15R (nt 1–20/nt 282–301), as published by Luongo et al. [4] and used to amplify the Fom301 marker, was previously confirmed to be specific to Fom [4]. A TaqMan-MGB probe, TDCpr1 (5’-6-FAM-TGCCACATGGACATTAT-MGB-NFQ-3’), was designed in conjunction with the Fom-specific primer sets Fa15F/Fa15R and TDCP2F/TDCP2R for PCR-based detection methods, following the sequence of Fom301 [4]. The combination of Fa15F/Fa15R primers and the TaqMan probe, TDCpr1, has been utilized for the molecular detection of Fom using both PCR [4,18] and Probe-qPCR [18]. However, this particular combination was found to be only suitable for detecting high levels of contamination (10% to 50%) in seeds [18], but not for low levels (0.25% to 5%). Therefore, in this study, the newly designed primers TDCP2F/TDCP2R were introduced, aiming to enable the molecular detection of low-level contamination in seeds using the TDCP2F/TDCP2R primers with the TDCpr1 system.

The primer set TDCP2F/TDCP2R and the TaqMan probe TDCpr1 were rigorously examined for characteristics, including their suitability with primer express 3.0, GC content, and melting temperature (Tm) value. Oligo 7 was employed to verify that there were no duplex formations or hairpin structures among the primers, probes, and DNA targets, assuring the reliability of the assays. As an internal control group for conventional PCR in this study, the conserved primer set ITS1/ITS4 was used to amplify~500-bp rDNA regions, including the ITS1, 5.8S rDNA, and ITS2 [29]. Conventional PCR and Probe-qPCR (with TaqMan probe TDCpr1) employing primer sets Fa15F/Fa15R and TDCP2F/TDCP2R were implemented for the comparison of the Fom detection assays. This allowed for subsequent comparative analyses with TDCP2F/TDCP2R, including sensitivity assessment, reproducibility validation, and seed health tests. The sequences of the primer sets ITS1/ITS4 (used as a PCR internal control), Fa15F/Fa15R, and TDCP2F/TDCP2R are listed in Table 7. Conventional PCR conditions and protocols for ITS1/ITS4 and Fa15F/Fa15R were adopted from White et al. [29] and Luongo et al. [4], respectively.

### 4.3. Sensitivity Assays

A thorough and comparative analysis of primer sensitivity with different templates was conducted, ensuring a rigorous examination of the performance characteristics of the selected primer sets. Three different templates, including genomic DNA (gDNA), cloned fragment DNA, and mycelia, were utilized in the primer sensitivity assays for comparative analysis. The gDNA was extracted following the method described by Lin et al. [42], then dissolved in 0.1× TE buffer (1 mM Tris-HCl and 0.1 mM EDTA, pH 8.0), and subsequently stored at −20 °C for use in future molecular detection assays.

The cloned fragment DNA was generated by amplifying a 301-bp DNA sequence using the primer set Fa15F/Fa15R. The resultant DNA was gel-purified, cloned into the pGEM^®^-T Easy vector (Promega Co., Madison, WI, USA), and sequenced. The calculation of the cloned fragment DNA’s copy number was based on the concentrations measured using a SPECTROstar Nano spectrophotometer (BMG Labtech, Ortenberg, Germany). This standard template was dissolved in 0.1× TE buffer and stored at −20 °C, ready for subsequent sensitivity assays. For sensitivity assays, serial dilutions were prepared from the gDNA of Fom (ranging from 10^6^ to 10^−1^ fg per reaction), the standard template (ranging from 10^9^ to 10^1^ copies per reaction), and mycelia of Fom without DNA extraction (ranging from 10^8^ to 10^0^ ng per reaction). These were then subjected to a comprehensive evaluation to assess the sensitivity of PCR-based methods.

### 4.4. Preparation of Artificially Inoculated Seeds

The melon seeds (*Cucumis melo* L. cv. Jill) used in this study were sourced from Known-You Seed Co. (Kaohsiung, Taiwan). Seeds were first surface-disinfested by immersing them in a 1% sodium hypochlorite (NaOCl, Clorox, Oakland, CA, USA) solution for 7 min, followed by rinsing in sterile water. They were then thoroughly dried on sterile filter paper within a laminar flow hood. To prepare seeds with different infection rates, a procedure adopted from de Sousa et al. [16] was used. Specifically, seeds were placed on PDA medium overrun with Fom hyphae and incubated at 25 °C for 7 days. A blotter test [10] confirmed a 100% infection rate among the inoculated seeds. These infected seeds were then proportionally mixed with non-infected melon seeds to produce batches with varying infection rates, including 25%, 10%, 5%, 2.5%, 1%, 0.5%, and 0.25%. After sampling, nucleic acids were extracted using the method described in Section 4.6 (DNA Extraction Methods) for subsequent molecular detection and analysis.

### 4.5. Preparation of Fom Pod-Inoculation

The peduncle inoculation procedure was performed on six melon plants within a greenhouse. The experiments were replicated three times. Briefly, a microconidial suspension (10^6^ spores per ml) was prepared, with 10 µL injected into the peduncle of an immature melon fruit. Upon maturing, the seeds were carefully collected from six independent melon fruits and incubated at a constant temperature of 26 °C for pathogen cultivation, to enhance pathogen detection. This involved six different seed incubation periods (0, 1, 3, 7, 14, and 21 days). The seeds were then dried on sterilized filter paper and assessed for Fom infestation through standard plating. Molecular detection assays were conducted on seeds from each incubation period, using two distinct DNA extraction methods as described in Section 4.6 (DNA Extraction Methods).

### 4.6. DNA Extraction Methods from Seed Sample

Two DNA extraction techniques, rapid DNA extraction, and spin column-based DNA extraction, were compared in this study. Seed samples (400 seeds per treatment) with differing artificial infection levels (0–25%) or various natural contamination stages were frozen in liquid nitrogen and finely ground using a mortar and pestle. Subsamples (100 mg each) were used for DNA extraction. Rapid DNA extraction involved placing the subsamples in a mortar, milling them in 400 µL of lysis buffer (100 mM Tris-HCl, 100 mM NaCl, 10 mM EDTA, 1% SDS, 2% PVP) and mixing in 1200 µL of eluting buffer (10 mM Tris-HCl, 1 mM EDTA, pH 8.0) [30]. The 5 µL gDNA-containing supernatant was then used for further molecular detection. The other protocol was based on spin column-based DNA extraction (Viogene genomic mini kit, Viogene-BioTek, Taipei, Taiwan), performed following the manufacturers’ instructions and the procedure described by Porebski et al. [43]. Genomic DNA was dissolved in 0.1× TE buffer (1 mM Tris-HCl and 0.1 mM EDTA, pH 8.0) and stored at −20 °C for future molecular assays. The experiments were replicated three times.

### 4.7. Molecular Detection Assays

Two molecular techniques (including conventional PCR and Probe-qPCR) were used with the primer sets Fa15F/Fa15R and TDCP2F/TDCP2R for seed health tests for comparisons. For conventional PCR analysis, each 20 μL PCR mixture contained the tested templates, 1× KAPA Taq ReadyMix (Kapa Biosystems., Inc., Wilmington, MA, USA), and 0.25 μM primers (Fa15F/Fa15R or TDCP2F/TDCP2R). The parameters for conventional PCR were denaturing at 94 °C for 3 min, followed by 30 cycles of denaturing at 94 °C for 30 s, annealing at 60 °C for 30 s, and polymerizing at 72 °C for 30 s, followed by a final extension at 72 °C for 5 min. All PCRs were performed using a T100^TM^ Thermal Cycler (Bio-Rad Laboratories., Co., Ltd., Hercules, CA, USA). PCR products were subjected to electrophoresis in 2.0% agarose gels. The agarose gel was visualized, photographed, and analyzed using Gel Doc^TM^ EZ Imager (Bio-Rad Laboratories., Co., Ltd., Hercules, CA, USA). For Probe-qPCR analysis, each 20 μL real-time PCR mixture contained the tested templates, 1X KAPA Probe FAST qPCR Kit Master Mix Universal (Kapa Biosystems., Inc., Wilmington, MA, USA), 0.25 μM primers (Fa15F/Fa15R or TDCP2F/TDCP2R), and the TaqMan-MGB TDCpr1 probe. The parameters for Probe-qPCR were 95 °C for 5 min, followed by 40 cycles of 95 °C for 10 s and 60 °C for 20 s (annealing and polymerizing). The Probe-qPCR analysis was monitored on a CFX96 Touch^TM^ Real-Time PCR Detection System (Bio-Rad Laboratories., Co., Ltd., Hercules, CA, USA).

### 4.8. Validations Data from the Molecular Detection Assays

To evaluate the reproducibility of the molecular detection systems, DNA was extracted from 100 mg subsamples of 400 seeds that were artificially inoculated with Fom at a 5% level. Two types of validation were carried out: intraday (duplicate PCR amplification of subsamples from 400 seeds) and interday (duplicate PCR amplification of the same DNA sample, performed at different time points). These validations were conducted following the methodology described by Skottrup et al. [36], and the reproducibility was assessed by calculating the coefficient of variation (CV) for duplicate PCR amplification of Fom-infected samples. This contamination was simulated by blending 20 Fom-inoculated seeds with 380 non-Fom-inoculated seeds. Two DNA extraction methods were employed for the analysis: a rapid DNA extraction technique and a commercial spin column-based procedure. The evaluation scheme encompassed analyses of four different samples at identical time points (intraday), along with four repeated analyses of the same samples at various time points (interday). The entire reproducibility assay was performed in triplicate. Following extraction, the DNA samples underwent both conventional PCR and Probe-qPCR analyses, and the coefficients of variation (CV) were subsequently computed.

Furthermore, to contrast the performance of the molecular detection protocols, critical metrics were evaluated, including precision (calculated as True Positives/(True Positives + False Positives)), recall (True Positives/(True Positives + False Negatives)), accuracy ((True Positives + True Negatives)/(True Positives + False Positives + False Negatives + True Negatives)), and F1 scores (the harmonic mean of precision and recall). These metrics were analyzed within the context of artificial pathogen contamination at levels ranging from 0% to 25%.

## 5. Conclusions

The comprehensive analysis from this study provides a coherent narrative that accentuates the efficacy, reliability, and practicality of the TDCP2 Probe-qPCR method for detecting Fom in melon seeds. These results hold significant scientific and practical value and are likely to shape both future research directions and real-world applications. The pronounced superiority of TDCP2 Probe-qPCR, especially when paired with column-based DNA extraction, suggests that it is an ideal tool that can profoundly impact the management and control of Fusarium-related diseases in melons, and potentially other crops. Additionally, this research introduces a rapid DNA extraction method for detecting Fom-inoculated seeds. This method, which only takes 5 min to obtain DNA samples, can be used as an alternative to traditional seed-borne pathogen diagnosis methods for Fom-inoculated seeds. The combination of the Fom rapid detection method, TDCP2 Probe-qPCR, and the DNA extraction method, can be employed as a swift and efficient tool for detecting Fom-inoculated seeds. Further potential research areas may focus on enhancing the method’s sensitivity and specificity for low-level Fom infections and exploring its applicability to other Fusarium species affecting different crops. We also aim to integrate these protocols into routine seed health testing procedures, potentially developing a standardized toolkit for global agricultural stakeholders. Moreover, the exploration of portable, field-based molecular diagnostics could facilitate real-time decision-making, reducing the impact of the disease on melon production worldwide.

## Figures and Tables

**Table 1:** Isolates of plant pathogens used in this study and their PCR specificity assay results.

Fungal Pathogens/Diseases	Code Number	Original Hosts	Geographic Location	PCR Specificity Assays		
				ITS1/ITS4 ^a^	Fa15F/Fa15R ^b^	TDCP2F/TDCP2R ^c^
*Fusarium oxysporum* f. sp. *melonis* (Fom)/Fusarium wilt of melon (FWM)	PM-TDC-F006	Melon (*Cucumis melo* L.) (M)	Kaohsiung city, Taiwan	+ ^d^	+	+
Fom/FWM	PM-TDC-F007	M	Kaohsiung city, Taiwan	+	+	+
Fom/FWM	PM-TDC-F008	M	Kaohsiung city, Taiwan	+	+	+
Fom/FWM	PM-TDC-F009	M	Kaohsiung city, Taiwan	+	+	+
Fom/FWM	PM-TDC-F010	M	Kaohsiung city, Taiwan	+	+	+
Fom/FWM	PM-TDC-F011	M	Kaohsiung city, Taiwan	+	+	+
Fom/FWM	PM-YJL-F053	M	Kaohsiung city, Taiwan	+	+	+
Fom/FWM	PM-YJL-F054	M	Kaohsiung city, Taiwan	+	+	+
*F. oxysporum* f. sp. *tracheiphilum*/Fusarium wilt of aspparagus bean	PM-YHL-F003	Asparagus bean (*Vigna unguiculata*)	Taichung city, Taiwan	+	−	−
*F. oxysporum* f. sp. *gladioli*/Fusarium wilt of gladiolus	PM-YHL-F019	Gladiolus (*Gladiolus alatus*)	Taichung city, Taiwan	+	−	−
*F. oxysporum* f. sp. *lactucae* (Fol)/Fusarium wilt of lettuce (FWL)	PM-YHL-F031	Lettuce (*Lactuca sativa* L.) (L)	Yunlin county, Taiwan	+	−	−
Fol/FWL	ATCC76616	L	Texas state, USA	+	−	−
*F. oxysporum* f. sp. *lilii*/Fusarium wilt of lily	PM-YHL-F035	Lily (*Lilium candidum*)	Taichung city, Taiwan	+	−	−
*F. oxysporum* f. sp. *lycopersici*/Fusarium wilt of tomato	PM-YHL-F042	Tomato (*Solanum lycopersicum*)	Taichung city, Taiwan	+	−	−
*F. oxysporum* f. sp. *niveum*/Fusarium wilt of watermelon	ATCC62940	Watermelon (*Citrullus lanatus*)	Texas state, USA	+	−	−
*F. oxysporum* f. sp. *cubense* (Foc)/Fusarium wilt of banana (FWB)	PM-YJL-F040	Banana (*Musa* sp.) (B)	Pingtung city, Taiwan	+	−	−
Foc/FWB	ATCC38741	B	Texas state, USA	+	−	−
Foc/FWB	ATCC76243	B	Texas state, USA	+	−	−
Foc/FWB	ATCC96285	B	Texas state, USA	+	−	−
*F. oxysporum* f. sp. *anoectochili*/Stem rot of anoectochilus	PM-YHL-F002	Anoectochilus (*Anoectochilus formosanus)*	Nantow county, Taiwan	+	−	−
*F. oxysporum* (Fo)/Endophyte	PM-YJL-F056	M	Taichung city, Taiwan	+	−	−
Fo/Saprophyte	PM-TDC-F012	Soils (S)	Kaohsiung city, Taiwan	+	−	−
*F. solani*/Saprophyte	PM-YJL-F055	S	Taichung city, Taiwan	+	−	−
*F. acuminatum*/Fusarium blight	PM-YHL-F018	Bermuda grass (*Cynodon dactylon* L.)	Taichung city, Taiwan	+	−	−
*F. verticilliodes*/Bakanae disease of rice	PM-YHL-F056	Rice (*Oryza sativa* L.)	Taichung city, Taiwan	+	−	−
*Colletotrichum gloeosporioides* (Cg)/Anthracnose of melon	PM-TDC-F013	M	Pingtung county, Taiwan	+	−	−
Cg/Anthracnose of melon	PM-TDC-F014	M	Pingtung county, Taiwan	+	−	−
*Alternaria* sp./Alternaria leaf spot of melon	PM-TDC-F016	M	Pingtung county, Taiwan	+	−	−

^a^ The conserved primer set ITS1/ITS4 was utilized to amplify and sequence the approximately 500-bp rDNA region, facilitating the identification of the internal transcribed spacers 1 (ITS1), 5.8S rDNA, and ITS2 in the fungal pathogens being tested. This approach aids in accurate identification and differentiation of fungal pathogens [29]. ^b^ The primer set Fa15F/Fa15R, specific to *Fusarium oxysporum* f. sp. *melonis* (Fom) race 2, was used in our specificity assays to confirm the results. This primer set was initially designed by Luongo et al. [4]. ^c^ Additionally, a novel primer set TDCP2F/TDCP2R, also specific to Fom race 2, was developed in this study to aid in further specificity testing. ^d^ + indicates a positive detection. − indicates a negative detection.

**Table 2:** Intraday and interday assays conducted for the molecular detection of 5% contamination by *Fusarium oxysporum* f. sp. *melonis* (Fom) in seeds.

Molecular Detection Assays	Detection Protocols			
	Intraday ^†^ Mean CVs ^§^ (%)		Interday ^‡^ Mean CVs ^§^ (%)	
	Commercial DNA extraction kit ^d^	Rapid DNA extraction method ^e^	Commercial DNA extraction kit	Rapid DNA extraction method
Conventional PCR ^‖^				
Fa15F/Fa15R	56.10 ± 7.14 ^e¶^	59.14 ± 1.79 ^ef^	52.75 ± 2.25 ^j^	42.31 ± 1.69 ^i^
TDCP2F/TDCP2R	15.59 ± 5.28 ^c^	63.77 ± 6.56 ^f^	13.92 ± 3.77 ^g^	59.39 ± 4.63 ^k^
Probe-qPCR				
Fa15F/Fa15R	3.87 ± 0.11 ^a^	ND ^††^	8.35 ± 5.18 ^def^	ND
TDCP2F/TDCP2R	0.78 ± 0.24 ^a^	1.74 ± 0.17 ^a^	1.50 ± 0.37 ^ab^	0.99 ± 0.03 ^a^

^†^ The analysis of four different samples was performed at the same time points. ^‡^ The analysis of the same samples was repeated on four different days. ^§^ Coefficients of variations (CVs) are calculated as the standard deviations divided by the mean intensity levels in gel electrophoresis in conventional PCR, and by the CT value for probe-qPCR, from each assay (*n* = 4), multiplied by 100%. ^‖^ Expected DNA bands were detected by the positive control ITS1/ITS4 [29] in all melon seeds tested. ^¶^ Means within columns followed by the same capital/lower case letters are not significantly different according to the LSD test at a 5% significance level. ^††^ ND denotes not detectable.

**Table 3:** Comparison of conventional PCR and Probe-qPCR methods for the detection of *Fusarium oxysporum* f. sp. *melonis* (Fom) in inoculated seeds using a commercial DNA extraction protocol.

Molecular Detection Assays	Ratio of Fom-Inoculated Seeds ^a^ (%)									
	25	10	5	2.5	1	0.5	0.25	0	P ^b^	N ^c^
Conventional PCR ^d^										
Fa15F/Fa15R	+ ^e^(9/9)	+(9/9)	+(9/9)	+(8/9)	+(7/9)	+(4/9)	+(3/9)	−(0/9)	+	−
TDCP2F/TDCP2R	+(9/9)	+(9/9)	+(9/9)	+(7/9)	+(7/9)	+(4/9)	+(3/9)	−(0/9)	+	−
Probe-qPCR										
Fa15F/Fa15R	+(9/9)	+(9/9)	+(9/9)	+(8/9)	+(8/9)	+(7/9)	+(7/9)	−(0/9)	+	−
TDCP2F/TDCP2R	+(9/9)	+(9/9)	+(9/9)	+(9/9)	+(9/9)	+(9/9)	+(9/9)	−(0/9)	+	−

^a^ Four hundred Fom-inoculated seeds were prepared by mixing specific quantities of Fom-inoculated seeds with non-Fom-inoculated seeds. ^b^ P denotes the positive control; 20 ng of Fom (TDC-F009) genomic DNA was used as the template. ^c^ N denotes the negative control; sterile ddH_2_O was added as the template. ^d^ Expected DNA bands were detected by the positive control using the ITS1/ITS4 primers [29] in conventional PCR for all melon seeds tested. ^e^ + indicates positive detection; − indicates negative detection.

**Table 4:** Comparison of conventional PCR and Probe-qPCR methods for the detection of *Fusarium oxysporum* f. sp. *melonis* (Fom) in inoculated seeds using a rapid DNA extraction protocol.

Molecular Detection Assays	Ratio of Fom-Inoculated Seeds ^a^ (%)									
	25	10	5	2.5	1	0.5	0.25	0	P ^b^	N ^c^
Conventional PCR ^d^										
Fa15F/Fa15R	+ ^e^(9/9)	+(9/9)	+(9/9)	+(8/9)	+(6/9)	+(2/9)	+(3/9)	−(0/9)	+	−
TDCP2F/TDCP2R	+(9/9)	+(9/9)	+(9/9)	+(8/9)	+(5/9)	+(2/9)	+(2/9)	−(0/9)	+	−
Probe-qPCR										
Fa15F/Fa15R	+(1/9)	−(0/9)	−(0/9)	−(0/9)	−(0/9)	−(0/9)	−(0/9)	−(0/9)	+	−
TDCP2F/TDCP2R	+(9/9)	+(9/9)	+(9/9)	+(9/9)	+(9/9)	+(9/9)	+(9/9)	−(0/9)	+	−

^a^ Four hundred Fom-inoculated seeds were prepared by mixing specific quantities of Fom-inoculated seeds with non-Fom-inoculated seeds. ^b^ P denotes the positive control; 20 ng of Fom (TDC-F009) genomic DNA was used as the template. ^c^ N denotes the negative control; sterile ddH_2_O was added as the template. ^d^ Expected DNA bands were detected by the positive control using the ITS1/ITS4 primers [29] in conventional PCR for all melon seeds tested. ^e^ + indicates positive detection; − indicates negative detection.

**Table 5:** Comparison of molecular detection protocols for *Fusarium oxysporum* f. sp. *melonis* (Fom)-inoculated seed samples (with various levels 0.25%, 0.5%, 1% 2.5%, 5%, 10%, and 25%) using column DNA extraction and rapid DNA extraction methods.

Protocols	Precision ^a^	Recall ^b^	Accuracy ^c^	F1 Score ^d^
Fa15F/Fa15				
Column DNA extraction protocol_cPCR	1.000	0.778	0.806	0.875
Column DNA extraction protocol_Probe-qPCR	1.000	0.905	0.917	0.950
Rapid DNA extraction protocol_cPCR	1.000	0.730	0.764	0.844
Rapid DNA extraction protocol_Probe-qPCR	1.000	0.016	0.139	0.031
TDCP2F/TDCP2R				
Column DNA extraction protocol_cPCR	1.000	0.762	0.792	0.865
Column DNA extraction protocol_Probe-qPCR	1.000	1.000	1.000	1.000
Rapid DNA extraction protocol_cPCR	1.000	0.698	0.736	0.822
Rapid DNA extraction protocol_Probe-qPCR	1.000	1.000	1.000	1.000

^a^ Precision = True Positives/(True Positives + False Positives). ^b^ Recall = True Positives/(True Positives + False Negatives). ^c^ Accuracy = (TruePositives + TrueNegatives)/(TruePositives + FalsePositives + FalseNegatives + TrueNegatives). ^d^ F1 Score is the harmonic mean of precision and recall; F1 score = 2 × precision × recall/(precision + recall).

**Table 6:** Comparison of published conventional PCR method (Fa15 cPCR) and TDCP2 Probe-qPCR methods for simulating the detection of *Fusarium oxysporum* f. sp. *melonis* (Fom) in pod-inoculated seeds.

Days after Incubation	Isolation	Molecular Detection			
		Commercial DNA Extraction Kit		Rapid DNA Extraction Method	
		Fa15 cPCR	TDCP2 Probe-qPCR	Fa15 cPCR	TDCP2 Probe-qPCR
0	− ^a^ (0/6)	−(0/6)	+(6/6)	−(0/6)	+(4/6)
1	+(4/6)	−(0/6)	+(6/6)	−(0/6)	+(5/6)
3	+(4/6)	−(0/6)	+(6/6)	−(0/6)	+(5/6)
7	+(4/6)	−(0/6)	+(6/6)	−(0/6)	+(5/6)
14	+(6/6)	−(0/6)	+(6/6)	−(0/6)	+(6/6)
21	+(6/6)	−(0/6)	+(6/6)	−(0/6)	+(6/6)

^a^ + indicates positive detection; − indicates negative detection.

**Table 7:** The primers list used in this study.

Primers Name	Target	Target Gene/Accession Number	Sequences (5’-3’)	Amplicon Size (bp)	Annealing Temperature	Reference
ITS1	All fungal	Internal transcribed spacer (ITS)/AY188919	TCCGTAGGTGAACCTGCGG (nt 1–19)	≈550	54 °C	[34]
ITS4	All fungal	ITS/AY188919	TCCTCCGCTTATTGATATGC (nt 525–544)			
Fa15F	*Fusarium oxysporum* f. sp. *melonis* (Fom)	Translation elongation factor 1-α (TEF-1α)/JN183059	TAGGGATGATAGCGGTCTGG (nt 1–20)	301	60 °C	[19]
Fa15R	Fom	Tef-1α/JN183059	GCTAGTTCGAGGCAATTGGA (nt 282–301)			
TDCP2F ^a^	Fom	Tef-1α/JN183059	TGGGATGGGAAATACCATGAC (nt 18–38)	114	64 °C	This study
TDCP2R ^a^	Fom	Tef-1α/JN183059	ACTGCCAGTTACGTGGCTTGT (nt 111–131)			

^a^ TDCP2F/TDCP2R primer set is able to amplify the 114-bp DNA fragment of Fa15_301_ (accession no. JN183059, nt 18–131).

## Data Availability

All the research data are shared in the manuscript.
